# Development of Guidelines for Implementing Digital Technology Into In-Home Aged Care: Mixed Methods Approach

**DOI:** 10.2196/86988

**Published:** 2026-09-16

**Authors:** Frances Batchelor, Susan Williams, Kerry Hwang, Baldwin Pok Man Kwan, Marissa Dickins, Tony Walsh, Helena Jakupovic, Kayla Lock, Tanya E Davison

**Affiliations:** 1 Clinical Gerontology, National Ageing Research Institute, 34-54 Poplar Rd, Parkville, 3052, Australia, 61 403996865; 2 Faculty of Medicine, Dentistry and Health Sciences, The University of Melbourne, Parkville, Australia; 3 Monash University, Clayton, Australia; 4 Research and Innovation, Silverchain, Osborne Park, Western Australia, Australia; 5 Federation University, Ballarat, Australia

**Keywords:** aged care, aging in place, digital technology, aged care workforce, older people, guidelines

## Abstract

**Background:**

In-home aged care is a vital part of Australia’s formal aged care system, offering essential support for older adults living in their homes. From 2023 to 2024, over 1.1 million Australians received in-home aged care, and this number is expected to grow. However, significant workforce shortages are currently occurring in the sector. One potential solution to workforce shortages is integrating technology into in-home aged care. Technology can support health monitoring, care coordination, emergency detection, social connection, and physical activity. Despite progress in residential aged care, community aged care lacks sufficient evidence on technological implementation.

**Objective:**

This study aims to develop guidelines for implementing digital technology for in-home aged care, considering the needs of clients, their families, care workers, and aged care providers.

**Methods:**

Guidelines were developed by a partnership consisting of a research institute and a national provider of in-home aged care. A 2-stage, mixed methods approach was used, integrating the data from a literature review, client and staff surveys, staff interviews, a co-design workshop, and case study to inform the guidelines’ content and format. Content was refined iteratively with aged care staff, older people, and researchers, using a participatory approach.

**Results:**

Seven key domains and 19 subthemes were identified as critical to the successful implementation of digital technology for in-home aged care. These included factors related to technology design, privacy and trust, training and support, organizational culture, client care, digital literacy, and perceived benefits. Each subtheme was accompanied by enablers, barriers, and practical implementation tips. The guidelines were refined through participatory methods and validated via a real-world case study involving smart glasses, demonstrating their applicability and relevance.

**Conclusions:**

The guidelines offer a comprehensive, evidence-informed framework to support aged care providers in implementing digital technology for in-home aged care. By addressing the unique challenges of in-home settings and incorporating the perspectives of clients, staff, and organizations, the guidelines aim to enhance care quality, client outcomes, and workforce sustainability. Further testing and refinement are recommended to strengthen client perspectives and evaluate broader applicability.

## Introduction

In-home aged care, also known as home-based aged care, is an essential component of the formal aged care system in Australia, providing critical support for older adults living in their own homes [1-3]. From 2023 to 2024, more than 1.1 million Australians received aged care in the home [3]. This number will continue to rise as the population ages, given the continued preferences of older people to remain living in their homes (and not move to supported accommodation) and the Australian policy reforms prioritizing home-based care [4,5]. Without timely and adequate access to in-home aged care, older people may experience functional decline; lower quality of life; mortality; and increased service utilization, including transition to residential aged care [6].

However, Australia and other high-income countries are facing significant shortages in the aged care workforce [7]. The shortage is a complex issue influenced by factors such as high workloads [8], relatively low pay for aged care workers, and high levels of casualization [9]. Addressing the shortages will require comprehensive strategies, including improved workforce planning, increased funding, and enhancing the status of this workforce [10]. However, other solutions are also needed.

One solution is to extend multidisciplinary approaches to in-home aged care by including technology-supported options. The potential roles of technology for in-home aged care are diverse and can include, but are not limited to, health monitoring, communication between care providers and those receiving care, emergency detection including fall detection, social connection and support, and supporting physical activity and exercise [11]. From the perspective of an aged care service provider, technology-enabled care could span the domains of care coordination, telehealth or remote delivery of care, rostering and scheduling, and staff training, among others [12].

However, while significant work has been done to investigate technological implementation in residential aged care, evidence related to in-home aged care remains scarce. Some of the reported barriers to the successful implementation of technology relating to aging in place include knowledge barriers and low digital literacy of many older people and in-home aged care staff, physical challenges in using technology, concerns about privacy, reduced involvement of end users in technology design, and difficulty in maintaining adherence [13,14]. The adoption of technology can also be influenced by factors such as cost, perceived value, and physical infrastructure to support technology [15].

To support successful implementation into in-home aged care, guidelines on how to effectively implement technologies are required. For in-home aged care, consideration needs to be given to all potential end users, including the individuals receiving home aged care services along with their care partners (eg, family and friends), care workers, and the aged care providers. In-home aged care is different from hospital or residential aged care settings because care is being provided in a person’s home or usual living environment rather than within a service or health provider’s premises.

We therefore aimed to address the lack of guidelines on digital technology implementation for in-home aged care by developing a comprehensive set of practical guidelines to enable aged care providers to implement technology, taking into consideration the needs and preferences of clients and their families, aged care workers, and aged care organizations. In this paper, we use the broader term “digital technology” to refer to digital health and care technologies relevant to in-home aged care, including technologies used for direct care delivery, monitoring, communication, care coordination, and workforce support. Where “digital health technology” is used, it refers to the technologies used to collect, share, or support health-related information and care.

## Methods

### Study Design

**Figure 1.**
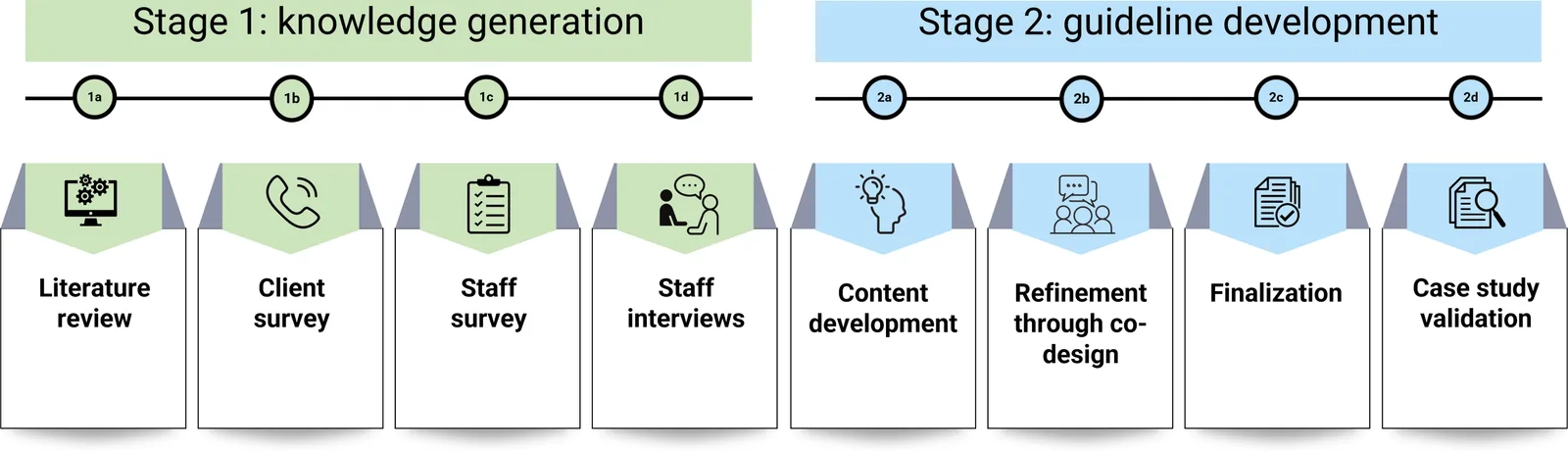
Study design: data sources and stages.

This study used a 2-stage approach to develop and refine the content and format of the guidelines (Figure 1). Each stage includes multiple components. In the first stage (knowledge generation), data were sourced from the results of a literature review, a survey of in-home aged care clients, and surveys and interviews with in-home aged care staff to inform the guidelines’ content. In the second stage (guideline development), a participatory design approach was used in which data from stage 1 was integrated to develop content and then further refined with aged care staff, consumers, and researchers in a participatory workshop. The draft guidelines were then tested using a real-life case study and the content and layout finalized.

### Procedures for Stage 1: Knowledge Generation: Sourcing Data for Guidelines’ Content

#### Component 1a: Literature Review

The literature review aimed to collate and synthesize existing evidence on enablers and barriers to the implementation of digital health technology for in-home aged care. Digital health technology was defined as the “systems, tools and services based on information and communications technology that can be used to treat patients and collect and share a patient’s health information” [16]. A narrative review with a systematic search strategy was conducted. CINAHL, EMBASE, PubMed, and PsychINFO databases were searched in March 2023. Conference abstracts, protocol abstracts, and papers not published in English were excluded, as were papers published more than 10 years prior to the search date due to the rapidly changing technology landscape. Search terms included those related to “aged care,” “ageing in place,” “home based care,” “home care,” “home aged care,” “Australia,” “aged care,” “technology/ies,” “enablers and barriers,” “factors,” “framework/s,” “digital health technology/ies,” “ICT, “robotics,” “implementation,” “ehomecare,” and “smart home technology/ies.” Multimedia Appendix 1 includes the search strategy. Each abstract was screened by 2 of the 3 researchers (KH, BK, SW). Disagreements were resolved by the third researcher. Further full-text screening was completed by 1 reviewer. Once the data extraction was complete, 1 researcher (BK) completed data quality analysis, using the Mixed Methods Appraisal Tool (MMAT) [17] for the nonreview papers, and completeness of reporting using the PRISMA (Preferred Reporting Items for Systematic Reviews and Meta-Analyses) guidelines and its extensions for the review papers [18]. Both informed the interpretation of the strength of the evidence; however, studies were not formally weighted nor excluded on the basis of the relevant score. Data were extracted using a customized data extraction template, including fields related to home care tasks, types of technology, outcomes, and enablers and barriers to technology implementation. To synthesize the enablers and barriers to the implementation of technology for in-home aged care, we used deductive thematic analysis [19].

#### Component 1b: Staff Survey

The aim of this component was to understand the digital readiness of in-home aged care staff, the potential for in-home aged care activities to be digitally enabled, and the enablers and barriers to using technology in delivering care. The survey was conducted with in-home aged care staff members across Australia involved in direct care. Table 1 and separate publications provide further details on the methodology and results [20,21].

**Table 1.** Stage 1: recruitment approaches, data collection, and data analysis.

Component	Recruitment approach	Data collection	Data analysis
1b: staff survey	Convenience sampling approach Eligible staff—in-home aged care workers and health professionals employed from a large Australian in-home aged care provider who provided in-home direct care in the preceding 12 months. Eligible staff identified by human resources data. Recruitment email sent to eligible staff. Additional recruitment through team meetings, newsletters, and follow-up emails.	Online survey (Qualtrics) Questions on personal technology use, attitudes toward technology (using the shortened Information Technology Attitude Scales for Health (shortened-ITASH) [22]) and digital health literacy (with the eHealth Literacy Scale [eHEALS] [23]). Implied consent was provided by completion of the survey.	Descriptive statistics (for categorical data—frequencies, percentages, and for continuous variables, means and SDs) were generated in Stata (version 18.0 BE; StataCorp). Open-ended questions were entered into NVivo and thematically analyzed.
1c: staff interviews	Purposive sampling approach. Eligible participants included current in-home aged care staff from a large Australian in-home aged care provider and were recruited either by opting into further research when completing the staff survey or by managers nominating employees to ensure a diverse range of roles and locations in the interview sample. Invitations were sent by email.	Semistructured interviews were conducted online using Microsoft Teams. Informed consent was provided by participants prior to the interview. The interviews were transcribed verbatim using Microsoft Teams.	Data were entered into NVivo and analyzed using applied thematic analysis. A codebook was developed based on the semistructured interview guide and further developed iteratively during analysis. Analysis was conducted by one researcher (BK), with a second (MD) conducting double coding of 20% (n=4) of the transcripts.
1d: client survey	A random sampling approach was used to recruit eligible individuals receiving in-home aged care services from a large Australian in-home aged care provider. Recruitment letters were sent to those individuals. Trained staff then called the individuals to recruit them for the study and complete data collection.	Questions specifically designed for the client survey were utilized during telephone-administered surveys. Informed consent was obtained from participants prior to the survey.	Descriptive statistics, including medians and IQRs for continuous variables and frequencies and percentages for categorical data, were generated in Stata (version 17.0 BE; StataCorp).

#### Component 1c: Staff Interviews

The aim of this component was to explore in further detail the digital enablement potential of common direct care tasks, benefits and drawbacks of technology use, and enablers and barriers to using technology in delivering care within in-home aged care. The interviews were conducted with in-home aged care staff members working in a variety of roles at a large, national aged care organization in Australia. See Kwan et al [21] for further details on the methodology and Table 1.

#### Component 1d: Client Survey

A national phone survey of in-home health and aged care clients to understand their ownership and use of technology was conducted by the aged care provider prior to the commencement of this project. The results pertaining to in-home aged care clients were used for the purpose of this study. See Table 1 and also Dickins et al for further details on the methodology and results (M Dickins et al, unpublished data, November 2022).

### Recruitment, Data Collection, and Data Analysis

#### Stage 1: Knowledge Generation

Information regarding recruitment, data collection, and analysis for components 1b-1d is included in Table 1.

Qualitative and quantitative data collection were conducted separately. The staff survey data informed one component of the staff interviews, that is, *Did the interviewee agree with the survey results of how different tasks could be assisted by technology were ranked*? All data were integrated in stage 2 by triangulation methods, bringing together data sources in a single dataset to cross-verify themes. The integration allowed conceptual thinking and multiperspectives to emerge.

#### Stage 2: Guideline Development

##### Component 2a: Content Development

Stage 2a involved iteratively collating and categorizing all data from stage 1 into themes and subthemes. These were directly used to develop the preliminary guideline content. In defining the domains and subthemes, data from each component of stage 1 was integrated equally with no preferential weighting or influence. We used a modified approach based on the framework method [24] for analyzing and collating data from multiple data sources, including from textual data and documents. Two (SW and KH) researchers familiarized themselves with the data from the literature review, surveys, and interviews and commenced coding into broad themes. The researchers then compared their initial findings. Codes were grouped into categories and applied to the data from each source. A spreadsheet was used to keep track of all categories, which were then grouped into themes and subthemes following further discussion between the researchers. Where overlaps or discrepancies existed, the researchers discussed the data and codes and mutually agreed on the category and/or theme. To minimize redundancy, while acknowledging categories would not be mutually exclusive, further iterations were made until no further combination or collapsing of themes or subthemes was possible. This final component was done by consensus between the researchers.

##### Component 2b Refinement

Stage 2b consisted of an online co-design workshop aimed at reviewing content, identifying areas for improvement, and making recommendations to enhance the guidelines. A purposive sampling approach was used to recruit participants. We aimed to have a broad representation across roles. Operational managers were contacted to nominate staff members from different in-home aged care roles to receive invitations to participate in the workshop.

The draft guideline content was shared with participants, and options for structure and format were presented by a facilitator. The facilitator’s role was to moderate the discussion, and feedback on the guidelines was agreed by consensus. The discussions and workshop feedback were captured by structured contemporaneous notes. These notes and feedback were reviewed by the project team after the workshop and incorporated where it improved clarity, practical usability, or alignment with the evidence generated in stage 1.

##### Component 2c: Finalization of Guidelines—Content and Format

Following the co-design workshop, we refined the content, wording, and format of the guidelines. We incorporated feedback from the co-design workshop and then reviewed and edited the guidelines text to reduce jargon. A technology implementation checklist was created as a companion document to the guidelines. We worked with a graphic designer to produce a draft of the guidelines to ensure the format and content were concise, appealing, and practical. The draft guidelines were provided to 4 experts and senior managers within the in-home aged care organization for review and feedback. This was a pragmatic review whereby the 4 experts and senior managers determined if the guidelines and checklist would meet their needs. They were asked to provide suggestions for improvement. The feedback received was then used in further iterations of the guidelines by incorporating the suggestions.

##### Component 2d: Case Study Field Testing

The finalized guidelines and associated checklist were field tested as a tool for use by aged care providers by applying them to a real-world implementation of smart glasses technology for people receiving in-home aged care in one business unit of the aged care provider. The field testing process involved seeking approval of the guidelines and checklist from those implementing a piece of technology. This was achieved by testing the guidelines and checklist as part of the implementation process. Smart glasses are devices with built-in cameras and live streaming capabilities. The plan was to implement this technology in a variety of clinical use cases such as remote specialist nursing wound review and remote allied health assessments in regional and remote areas. The guidelines and checklist were provided in a working session between the smart glasses implementation team and researcher BK, with instructions provided for use of the guidelines and checklist. The implementation team used the guidelines and checklist to analyze potential enablers and barriers to introducing smart glasses into the workflow and developed an implementation plan.

The feedback received from the implementation plan was provided to BK and enabled further refinement of the guidelines to improve the applicability and usability for the in-home aged care context.

### Ethical Considerations

Ethics approval for the client survey, staff survey, and interviews was obtained from the Silver Chain Group (SCG) Low and Negligible Risk Ethic Review Panel (LNR; LNR EC Project Numbers RG-054 and RG-061). Informed consent was obtained from all participants, as per the approved LNR protocol. A data management plan was approved, meeting all privacy and confidentiality requirements. Survey data were deidentified; interview data transcripts were analyzed in reidentifiable form, with participant codes kept separate from the anonymized transcripts as per protocol. No compensation was provided to participants.

### Reflexivity or Positionality Statement

FB, SW, KH, and KL are researchers at a national aging research institute, with FB and SW previously working as physiotherapists in health and aged care settings. MD, BK, and TED are researchers embedded within an in-home aged care provider, with TED also being a clinical psychologist. BK is also a physiotherapist working within in-home aged care and hospital settings, with qualitative and quantitative methodology training. TW and HJ are people with lived experience of aged care at home. BK, MD, and TED are employees of the in-home aged care provider at the time of the research. This was a deliberate choice to ensure the inclusion of the views of frontline aged care staff in shaping the direction of this study and ensuring its relevance and usability for aged care organizations. The majority of the qualitative data collection was conducted by SW, and the co-design workshop was co-facilitated by FB and an experienced workshop facilitator from the aged care provider to manage potential biases in data collection. The analysis of the survey and interview data and case study testing were led by BK to incorporate the views of frontline staff in interpreting the results. However, BK discussed the analysis and interpretation with the research team throughout the research process to ensure potential biases were discussed and managed.

## Results

### Data Sources

#### Literature Review

**Figure 2.**
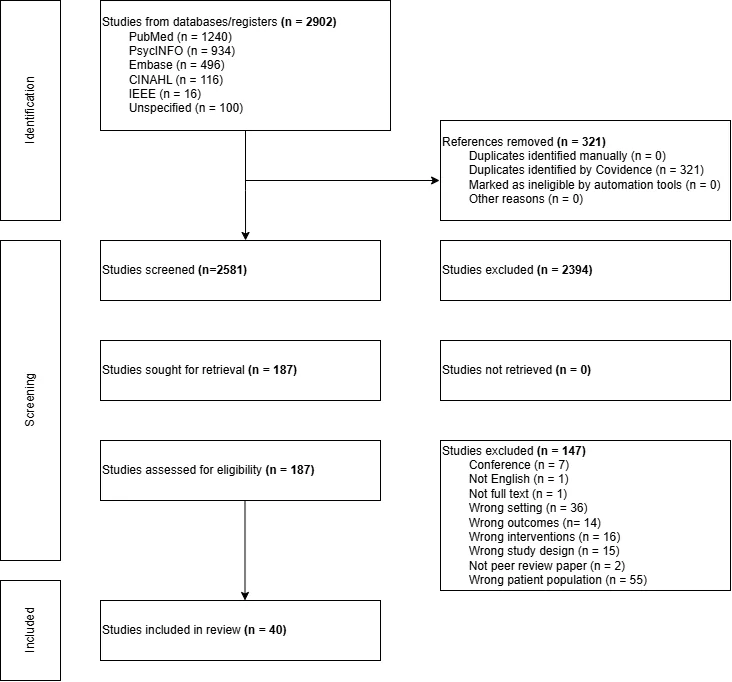
Literature review flow diagram.

The search resulted in 2902 references. After screening of abstracts and full text, a total of 40 studies were included based on the search criteria and were included in the review (Figure 2). In terms of quality, most nonreview papers had an overall Mixed Methods Appraisal Tool score of 5, indicating all the quality criteria were met. For the systematic reviews, scores ranged between 19 and 29 (out of 38), and for the 2 scoping reviews, scores were 14 and 18 (out of 22). From the deductive analysis, the enablers and barriers to technology implementation could be broadly categorized under “people,” “technology,” and “organizational” themes.

In relation to *people*, major barriers associated with implementing technology included the perceived increased workload associated with the introduction of new technology, unfamiliarity with technology, and resistance to change. The level of trust in technology, privacy and security considerations, and the relationship between client and aged care worker were all seen as impacting implementation. Other factors impacting implementation included a person’s (home care client or aged care worker) technological competency and skills, as well as their attitudes toward and perceptions of technology. Financial reimbursement for using technology was found to be an enabler.

Under the *technology* theme, technical issues were identified as barriers to implementation of technology into in-home aged care. This included device and system limitations (eg, poor video quality or absence of essential functions), and lack of system integration. Conversely, technologies that were perceived as simple and user-friendly were described as key enablers of successful implementation. Ease of use was seen as particularly important in the context of aged care, where both staff and clients may have varying levels of digital literacy. The perceived benefits or usefulness of technology also plays a critical role in adoption. High levels of perceived usefulness were associated with greater acceptance and integration into care. A lack of staff involvement in the design and implementation process emerged as another barrier. When staff were not engaged in shaping the end user experience, technical issues were more likely to persist.

The key factors influencing implementation at an *organizational* level included organizational culture and leadership, collaboration, infrastructure and resourcing, and financial considerations. A supportive organizational culture was identified as a key enabler, along with strong internal processes, effective leadership, and presence of champions. Ensuring effective partnerships with all stakeholders involved in care was seen to enhance integration and sustainability of technological solutions. Organizational infrastructure, including human resources, was seen as a critical factor in implementation. Barriers included lack of senior management engagement, understaffing, and inadequate IT infrastructure and technical support availability.

#### Staff Survey

A total of 267 Australian in-home aged care staff from a range of service roles including allied health professionals (n=31, 12%), care aides or assistants (n=43, 43%), nurses (n=5, 2%), domestic assistants (n=64, 24%), care managers or coordinators (n=31, 12%), and team managers (n=21, 8%) were recruited [20]. The response rate was unable to be calculated as it was not possible to determine how many people received the invitation to participate, given staff turnover and currency of correct email addresses. The participants were 91% female; 64% were aged between 45 and 64 years; 57% employed part time; 73% were metro based, 12% regional, 28 % rural, and 4% working remotely (sum is greater than 100% as some staff worked across 2 or more geographic settings); 26% had a bachelor’s degree or higher; and 49% had worked in in-home aged care for less than 5 years. They completed the survey on personal technology use, attitudes toward technology, and digital health literacy. The key findings relevant to the guidelines were related to digital readiness as both an enabler and barrier to technology implementation. Digital readiness refers to individuals’ preparedness and motivation for digital technology, which may influence digital technology use that is unrelated to actual opportunity. This is in comparison to digital literacy, which is defined as the capabilities and skill set in knowing how to use digital technology. In addition, confidence and acceptability determine how a new technology may succeed or fail based on user belief in their ability to use it. To generate subgroups of low, moderate, and high digital readiness, latent profile analysis (LPA) was conducted, using the participants’ personal use of digital technology, and the shortened Information Technology Attitude Scales for Health (shortened-ITASH) and eHealth Literacy Scale (eHEALS) scores as indicator variables [25]. The majority (n=235, 88%) of the participants demonstrated moderate-to-high digital readiness, suggesting that most of the workforce is capable of adopting technology with appropriate support [20]. There was also a smaller cohort of highly digitally ready (n=65, 24%) staff members, who may have a potential role as technology champions or buddies [20]. A notable minority (n=32, 12%) had lower digital readiness, with lower digital health literacy and less positive attitudes toward technology [20]. This highlighted the need to provide targeted support to those who may be less able or inclined to engage with new technologies, through tailored digital education and support strategies, which may be critical steps in enabling technology implementation. Furthermore, key enablers and barriers for incorporating digital technology into care processes identified by participants included the presence or lack of having someone to help troubleshoot if or when the technology does not work and having access to reliable technology [21].

#### Staff Interviews

A total of 18 interviews were completed with current in-home aged care staff, at which point interview saturation was reached as assessed by no further new information or themes emerging. Most (n=11, 61%) staff worked in metropolitan areas, and slightly more than half (n=10, 56%) of the staff interviewed had 5 or less years of experience working within in-home aged care [21]. Interview participants reinforced the findings of the staff survey that some direct care tasks could be digitally enabled, but this varies across roles. Communication was a task that was rated highly as likely to be able to be digitally enabled in the staff survey [21]. However, interview participants found that communication tasks with team members and clients were currently digitally enabled but not necessarily successfully in many situations. Apart from the barriers and enablers from the staff survey, interview participants also raised several additional barriers and enablers, such as the impact of data connectivity on technology use [21]. The staff interviews themes and subthemes are reflected in the guidelines.

#### Client Survey

Overall, 359 home aged care recipients participated in the client survey, with the majority being female (n=266, 74%) and aged 75 or older (n=291, 81%; M Dickins et al, unpublished data, November, 2022). A fifth (n=72, 20%) of the sample had a bachelor’s degree or higher. Most participants (n=320, 89%) had at least 1 type of technology available at home, with mobile phones being the most common type (n=258, 72%) followed by internet or Wi-Fi (n=205, 57%). The median number of types of technology at home was 3 (IQR 1-5) in this cohort. Only a third (n=129, 36%) of the participants surveyed used technology for health-related reasons. Of these, the most common reason to use health-related technology was to monitor their health issues (n=176, 49%). Most participants who reported they would not use health-related technology in the future felt that they had no need for it or felt that it was unnecessary (n=223, 62%; M Dickins et al, unpublished data, November, 2022). The results indicated that in-home aged care recipients used technology at home but at a lower proportion than the general population. The difference in technology usage may be a barrier to technology implementation within in-home aged care service delivery.

#### Co-Design Workshop

The workshop, facilitated by members of the project team, was a 2-hour online session with 10 staff (4 care workers, 40%; 1 allied health professional, 10%; 2 care coordinators, 20%; and 3 team leaders, 30%) from across the collaborating in-home aged care provider. TW, who is part of the research team as a consumer representative, also participated in the workshop to add the consumer perspective to the discussion.

The co-design workshop provided preliminary validation of the themes identified to date, confirmed the language used in the guidelines, identified practical strategies to implement, and generated additional enablers and barriers to the implementation of technology. For example, participants discussed the importance of having back-up systems available when technological issues occur, which was added into the guidelines as a practical strategy for the implementation team. This component was instrumental in confirming the themes of the draft framework but also in generating the practical strategies to support each of the implementation factors.

#### Case Study Field Testing

As a result of the case study field testing working session, two additional strategies to improve the implementation of the smart glasses were identified: (1) ensuring the benefits of smart glasses have been communicated to stakeholders of the implementation and (2) providing training for the workforce, especially in rural and remote areas, to ensure the training is relevant for their local context. Feedback regarding the guidelines and checklist included suggestions for improving the instructions to users of the guidelines, and the layout and accessibility of the checklist.

### Guidelines—Final Content

Following the collation and analysis of all data sources, 7 overarching domains and 19 subthemes that should be considered for successful implementation of digital technology were identified (Table 2). For each subtheme, enablers, barriers, and practical tips for implementation were also identified to help providers systematically approach technology implementation. Examples for 2 subthemes are provided in Table 3, and an example of the pathway to the development of a subtheme is provided in Table 4.

In addition to the main domains and subthemes, the guidelines also provided information about how to use the guidelines, including the need to critically evaluate whether technology is the best solution to achieving a specific outcome and the need for careful consideration of those impacted by the introduction of a technological solution. Suggestions about how to use the implementation checklist (Multimedia Appendix 2) were also provided, including the potential benefits of using the checklist at multiple time points during implementation.

**Table 2.** Guideline main domains and subthemes (source: Batchelor et al [26]).

Domain	Subthemes
Technology design factors and features	1.1 Familiarity and ease of use 1.2 Technical issues 1.3 Infrastructure 1.4 Interoperability
Privacy, security, and trust	2.1 Client privacy 2.2 Reliability and trust
Training and technical support	3.1 Training for staff 3.2 Training for clients 3.3 IT technical support
Organizational design and culture	4.1 Digital readiness 4.2 Confidence and acceptance of technology 4.3 Impacts on workload 4.4 Communication
Aspects of client care	5.1 Client preferences for technology 5.2 Suitability of technology for enhancing care
Digital literacy	6.1 Staff self-efficacy and preferences for technology 6.2 Client and staff digital literacy
Perceived benefits of technology	7.1 Usefulness 7.2 Awareness of benefits

**Table 3.** Examples of enablers, barriers, and practical tips for two subthemes: (1) client preferences for technology and (2) reliability and trust (source: Batchelor et al [26]).

Enablers	Barriers	Practical tips
5.1 Client preferences for technology (description: In-home aged care clients have diverse preferences for technology, based on their experience with technology)		
Some clients are enthusiastic about technology and are willing to adopt and learn how to use technology that may be of benefit to them. Some types of technology have greater uptake among clients. For example, many in-home care clients prefer using tablets to computers or smartphones.	Use of technology may be seen to depersonalize care. Many clients prefer face-to-face rather than remote contact. One-off or insufficient screening of preferences for clients may fail to identify the level of readiness for technology.	Highlight the introduction of technology that improves delivery of timely, individualized, and quality care. Avoid assumptions about clients’ attitudes, abilities, and competency toward technology, and instead, assess client preferences for technology to identify suitable technological options. Identify suitable “early adopters” to trial the technology. Ensure clients’ technology choices are clearly indicated in the client record and create alternative pathways for clients to receive care that are not reliant on technology. For example, ensure paper-based copies of forms are available in addition to electronic versions.
2.2 Reliability and trust (description: Unreliable technology compromises the delivery of care)		
Testing of technology in a variety of scenarios or situations prior to implementation can help to ascertain reliability. Using familiar technology can instill trust for clients, staff, and organizations.	Unreliable technology can inhibit trust in technology for improving the delivery of care.	Common issues that may impact care and reduce trust in technology include reduced internet connectivity, poor battery life, and slow processing speed. Test (and retest) new technology within the real-world of in-home aged care as part of the implementation phase.

**Table 4.** Example analytic pathway: organizational design and culture (subtheme “Impacts on workload”).

Analytic step	Example	Implication for guidelines
Evidence	Peer-reviewed literature identified “increased workloads” as a barrier, noting that new technology can increase workload because staff need time for implementation, orientation, and troubleshooting, while also continuing usual care and supporting clients to use the technology.	This provided evidence that workload impact was not just a technical issue but an organizational implementation issue.
Additional supporting information from interviews or surveys	“Time constraints” identified as a barrier, noting that organizations or staff with busy workloads are time poor, which can create constraints in implementing technology.	This reinforced the code that workload and available time affect readiness and implementation capacity.
Initial codes or categories	Workload capacity, time factors, staffing, piloting, and staged rollout.	These related codes were grouped because they all concerned the organizational capacity required to absorb, test, and embed new technology into routine work.
Subtheme	Impacts on workload subtheme, with enablers and barriers drawn from evidence	The codes were collapsed into a final subtheme focused on workload impact, reflecting both barriers and implementation strategies.
Final domain	Organizational design and culture	The subtheme was placed within this domain because workload impact is shaped by organizational planning, staffing, workflow design, training time, and implementation support.

## Discussion

### Principal Findings

In this study, user-friendly practical guidelines specific to the implementation of digital technology in the Australian in-home aged care setting were developed. A multicomponent, participatory approach was used to ensure that the guidelines were evidence-informed, grounded in the real-world, were practical, and recognized the unique nature of in-home aged care. The evidence informing the guidelines came from stage 1 of this study. The results of the stage 1 components supported the complexity of implementing technology into in-home aged care services and workflow. The enablers and barriers to implementation were identified in all data sources—published literature and clients and staff of a large in-home service provider. The guidelines are freely available for organizations to support the implementation of technology and increase the likelihood of successful integration. Without a systematic approach, attempts to incorporate technology into aged care settings are at risk of failing. Offering practical tips to enhance enablers and to minimize barriers was an important aspect to incorporate into the guidelines.

The guidelines offer specific implementation strategies addressing client- and staff-specific factors that may impede implementation. For example, from the client survey data, nearly two-thirds of older people surveyed did not feel technology was needed or necessary and, hence, do not use technology for their care. In response, practical implementation strategies that could address these factors were included in the guidelines, including educating staff and clients about the potential benefits of technology and linking it to their specific needs, and assessing the end users’ digital literacy levels for suitability to use technology. These strategies recognize that, at the individual level, staff and client digital literacy, confidence, and perceived need influence readiness to engage with technology.

Acknowledging that the 7 main domains are not unique to in-home aged care and have been identified in other contexts for digital technology implementation, these guidelines nonetheless provide a novel and important framework that provides details specific to in-home aged care, an area of increasing importance in Australia and throughout the world. The in-home aged care setting differs from other settings, such as residential aged care or hospital, in that the delivery of care is decentralized and occurs in a person’s home without the physical, digital, or data infrastructure or ecosystems associated with physical institutions. Staff travel between clients and mostly work on their own; therefore, they may experience digital connectivity issues, and their access to IT or other support is more limited when compared to care staff who work in hospitals or in aged care facilities. Clients may also have greater reliance on staff for technical support.

There were some similarities between staff and clients in relation to enablers and barriers to technology implementation. Based on the client survey, we identified that care recipients had good access to technology; however, support systems may not be as easily accessible. Technology and peer support are more readily available to staff through their organizations, whereas older people receiving in-home aged care often rely on the in-home aged care staff for their technology support (especially if family are not available or not close by). This highlights the interpersonal nature of technology implementation in in-home aged care, where relationships between clients, family carers, and care workers shape trust, communication, and willingness to trial technology.

The guidelines also recognize that the implementation of technology is multilayered, with multiple potential end users. The guidelines take into consideration that in-home aged care involves clients, their family members or other unpaid carers, and the aged care workforce, with care encounters occurring within a home environment. At the organizational level, leadership, training, resourcing, workflow design, and implementation support influence whether technology can be embedded into routine practice. The practical tips and checklist are aimed at aged care providers implementing new technology but can also be used by any of the potential end users to identify areas for improvement for better implementation or integration. These levels are interdependent; for example, a client’s willingness to use technology may be undermined if staff lack confidence, if technical support is unavailable, or if connectivity is unreliable.

### Limitations

This study has several limitations. First, while the guidelines were developed using a robust, multicomponent participatory process, the client perspective was not directly captured through interviews, potentially impacting the comprehensiveness of the guidelines. Although a client survey was conducted and consumers were represented on the research team, richer qualitative insights from clients may be needed to ensure the guidelines fully reflect their lived experiences and preferences, particularly around aspects of privacy, trust, preferences for care, and perceived benefits. Future work should include direct engagement with clients through interviews or focus groups to strengthen this component. This additional evidence may require further refinement of the guidelines. Also, family carers were not directly included in surveys, interviews, or workshops, and their perspectives would strengthen the guidelines if included.

Second, the guidelines were field tested using a single case study involving smart glasses. While this provided valuable insights into real-world applicability, broader testing across different technologies and care contexts is needed to assess generalizability and scalability. Additional trials in diverse settings and with varied technologies will help refine the guidelines and ensure their relevance across the sector.

Third, the study was conducted in partnership with a single aged care provider. Although this enabled deep engagement and practical relevance, perspectives from other providers, including smaller organizations and those operating exclusively in rural or remote areas, may differ. In addition, the resources available to a large national provider, including IT infrastructure, workforce development capacity, and dedicated implementation support, may not be available to smaller providers or organizations operating in more geographically isolated settings. Expanding the scope to include a wider range of providers could enhance the inclusivity and applicability of the guidelines. Finally, while the guidelines aim to be practical and user-friendly, their implementation may be influenced by organizational readiness, resource availability, and staff capacity. Ongoing evaluation and support will be essential to ensure successful uptake and sustained use. This could be conducted with the current single aged care provider and also in future studies evaluating the framework for additional implementation outcomes such as acceptability and feasibility. Future testing should also examine how the guidelines could be adapted for providers with different funding models, workforce profiles, and digital infrastructure.

The limited financial and other resources for the project led to data collection and analysis methodological choices, which could be strengthened in future work. For example, due to time and cost constraints, only 1 reviewer conducted the full-text screening in the literature review. Convenience or purposive sampling for the staff surveys and co-design workshop may have led to sample bias.

Finally, this study focused on the development of guidelines and did not evaluate implementation outcomes such as adoption, fidelity, cost-effectiveness, or impact on care. Future research should examine whether the use of these guidelines improves implementation processes across different types of technology, provider types, and client groups.

### Comparison With Prior Work

The findings align with previous work related to technology adoption by older adults. An integrative systematic review on the facilitators and barriers to technology adoption by community-dwelling older adults with chronic diseases identified 5 domains: demographic or socioeconomic, health-related, dispositional, technology-related, and social factors [27]. The findings from the review are highly relevant to in-home aged care due to the high prevalence of chronic diseases in people receiving in-home aged care; however, our work extends beyond these factors to also include the factors relevant to the implementation within aged care, rather than just the individual-level factors. Another review outlines the categories of factors affecting the implementation of health care technologies relevant to older adults, including factors that may have more or less applicability in different settings [28]. Factors such as user and caregiver attitudes and perceptions, organizational and governance factors, and contextual factors are also aligned with our findings, but our work included significant stakeholder input to support preliminary validation of the guidelines and ensure they have specificity and applicability to the in-home aged care sector.

Broad implementation science frameworks provide information on ways including enablers and barriers to ensure successful uptake of new programs and products. Our pragmatic, practical guidelines are specific to in-home aged care. The enablers and barriers to technology implementation within in-home care are similar to other aged care settings. Many Australian aged care organizations deliver both residential aged care and in-home aged care services, and the guidelines specific to the in-home aged care setting may be applicable to residential aged care settings. For example, staff attitudes and negative perceptions of technology have been reported in residential aged care settings [29], and cost and lack of skills have also been reported as barriers [30,31]. One study examining technology-mediated care in residential aged care found that staff identified resource constraints, client challenges, limited staff and organizational support, and family resistance as barriers to technology implementation [32]. Another systematic review of the enablers and barriers to the implementation of socially assistive humanoid robots in health and social care found that technical problems and lack of perceived usefulness of the device were barriers to robotic technology implementation [33]. Use of change agents in residential aged care facilities has been found to be an important factor for technology implementation for support, training, and troubleshooting [34]. However, as mentioned above, factors that are unique to in-home aged care were not included in these past papers.

### Conclusions

The guidelines provide a comprehensive, evidence-based tool for aged care providers who are wanting to implement digital health technology in their organizations with their clients. This is the first step in tool development—broader testing across multiple providers, technologies, and client groups is still needed. By addressing the specific needs and challenges of the in-home aged care sector, the guidelines aim to improve the quality and efficiency of care for older people and potentially make aged care services more responsive, personalized, and sustainable.

## Supplementary material

10.2196/86988 Multimedia Appendix 1 Literature review example search strategy.

10.2196/86988 Multimedia Appendix 2 Implementation checklist.

## Abbreviations

eHEALS: eHealth Literacy Scale
LNR: Low and Negligible Risk
LPA: latent profile analysis
PRISMA: Preferred Reporting Items for Systematic Reviews and Meta-Analyses
SCG: Silver Chain Group
shortened-ITASH: shortened Information Technology Attitude Scales for Health
