# Supplementary material for: Development of Guidelines for Implementing Digital Technology Into In-Home Aged Care: Mixed Methods Approach
Source: JMIR Form Res. 2026 Sep 16;10:e86988. doi: 10.2196/86988 (PMC13581284; doi:10.2196/86988)
Supplement: Supplementary file 1 [file formative-v10-e86988-s002.pdf]

Implementation Checklist

1. What gap is being addressed?

2. How will the identified technology address this gap?

3. Who are the key stakeholders involved in successful implementation of this solution?

Don't forget clients, families and all staff who will interact with this solution.

4. What is the risk of...

a. Implementing the technology?

b. Not implementing the technology?

5. What will success look like?

i.e. how will you evaluate the implementation of this technology? What will you measure to demonstrate this success?

a. Clients/Carers:

b. Frontline staff:

c. Management:

d. Other relevant stakeholders:

© NARI 2024

6. List the enablers, barriers and strategies related to:

1. Technology Design Factors and Features

- Design features
- Technical issues
- Infrastructure
- Familiarity and interoperability

Enablers	Barriers	Strategies

2. Privacy, Security and Trust

- Client privacy
- Reliability and Trust

Enablers	Barriers	Strategies

© NARI 2024

3. Training and Technical Support

- Training for staff
- Training for clients
- IT technical support

Enablers	Barriers	Strategies

4. Organisational Design and Culture

- Culture
- Digital readiness
- Confidence and acceptance of technology
- Impacts on workload
- Communication

Enablers	Barriers	Strategies

5. Aspects of Client Care

- Client preferences for technology
- Suitability of technology to enhance care

Enablers	Barriers	Strategies

© NARI 2024

6. Digital Literacy

- Staff self-efficacy and preferences for technology
- Level of digital literacy

Enablers	Barriers	Strategies

7. Perceived Benefits of Technology

- Usefulness
- Awareness of benefits

Enablers	Barriers	Strategies

© NARI 2024
