# Supplementary material for: Development of Guidelines for Implementing Digital Technology Into In-Home Aged Care: Mixed Methods Approach
Source: JMIR Form Res. 2026 Sep 16;10:e86988. doi: 10.2196/86988 (PMC13581284; doi:10.2196/86988)
Supplement: Supplementary file 2 [file formative-v10-e86988-s001.docx]

## Supplementary Information

Example search strategy

| Line | Search terms |
| --- | --- |
| 1 | (MH "Home Care Services+") OR (MH "Community Health Nursing") OR (MH "Health Services for the Aged") OR "home care" OR "home nurs*" OR "district nurs*" OR "community based care" OR "community health care" OR "care in the home" OR "aged care" OR "Senior Care" OR "Homecare" OR "elder care" OR "elderly care" |
| 2 | (MH "Ageing") OR (MH "Gerontology") OR (MH "Geriatrics") OR (aged or elder* or ag?ing or senior* or pension*) OR ((old* or senior*) adj (person* or people* or adult* or citizen*)) |
| 3 | "Technology" OR "smart home technolog*" OR "robot*" OR "smart" OR "virtual reality" "technolog*" OR "internet of things" OR "technolog* device" OR "telehealth" OR "digital health technolog*" OR "homecare technolog*" OR "home care technolog*" |
| 4 | 1 AND 2 AND 3 |
| 5 | "assistive technolog*" OR "assistive devices" OR "devices" OR "adaptive technology" |
| 6 | "enablers" or "facilitators" or "factors" or "barriers" or "implement*" or "framework" |
| 7 | #4 NOT #5 AND #6 |
| 8 | 2013-2023 |
